# Ecology, behavior and bionomics: functional response *of Heterotermes tenuis* Hagen (Insecta: Blattaria: Isoptera: Rhinotermitidae) in forests of the Colombian Orinoquía

**DOI:** 10.1186/s40850-023-00184-7

**Published:** 2023-10-26

**Authors:** Luis Ricardo Salazar-Salazar, Olga Patricia Pinzón-Florian

**Affiliations:** Universidad Distrital Francisco José de Caldas Bogotá, Bogotá, Colombia

**Keywords:** Functional diversity, Traits functional, Homogenization, Environmental filters

## Abstract

**Background:**

Land use intensification may affect diversity, abundance, and functional morphological traits (FMT) related to dispersal, food acquisition, digestion, and nesting in some insects, possibly impacting their ecological role. Most studies of termites on the effects of afforestation focus on diversity and abundance, but changes in FMT have yet to be studied.

**Aim:**

To better understand the response mechanisms to land use intensification, we compared the FMT of the worker and soldier caste of *Heterotermes tenui*s among *Pinus caribaea* plantations of four different ages and gallery forests of the Colombian Orinoquía.

**Methodology:**

We measured thirty-eight FMTs in the worker and soldier castes of *H. tenuis* from gallery forests and pine plantations. Then, we used a Community-Weighted Mean (CWM), a PERMANOVA, and a nonmetric multidimensional scaling (NMDS) to estimate the possible effect of land use type on the FMT of both castes. We selected the FMTs with the lowest intraspecific coefficient of variation (CV) from each caste to compare their size among the land use types and pine plantation ages.

**Results:**

Land use type had a more significant impact on the FMT size of pine plantation workers than the age of the afforestation. FMT of the worker caste tends to be larger in gallery forests than in pine plantations, while the results were inconclusive for soldiers.

**Conclusion:**

The results suggested a homogenization mainly of the feeding FMT of the worker caste of *H. tenuis* in pine plantations associated with the increase in the softwood food resource of *P. caribaea.*

**Supplementary Information:**

The online version contains supplementary material available at 10.1186/s40850-023-00184-7.

## Background

Epigeal and subterranean soil arthropods are considered bioindicators due to their diversity, participation in various ecosystem processes, and sensitivity to land-use changes [1, 2]. Most studies of the effect of changes in land use on edaphic fauna focus on the taxonomic diversity and abundance of particular taxa, such as carabids [3], springtails [4] and termites [5, 6]. However, ecosystem processes also depend on organism functional responses [7], such as changes in FMT related, for example, to the tasks of dispersal, food acquisition, digestion, and nesting [1, 8, 9].

An important fact to consider in studying body size traits is the need to estimate intraspecific variability (IV). Ignorance of IV can mask or overestimate interspecific effects and lead to false conclusions about the mechanisms that determine the patterns of diversity, habitat filtration, and habitat differentiation, among others [10, 11].

The most studied functional traits in insects are trophic level, dispersal capacity, voltinism, and body size [12]. The trophic level has been studied in Coleoptera [13], Hemiptera [14], and termites [15], while dispersal capacity has been studied in ants [16, 17]. The study of body size allows the comparison of the responses of different organisms and communities to disturbances and the possible consequences in the habitat due to these modifications [18]. Specifically, studies have focused on Coleoptera due to their taxonomic diversity, wide distribution, and sensitivity to land use [19].

Changes in land use can influence taxonomic diversity [20, 21], the frequency of trophic groups [15, 22], and the functional morphological traits (FMT) of termites [21]. Changes in FMT have unknown consequences on the ecosystem services related to decomposition, bioturbation, and favoring of the local diversity that they contribute to the ecosystems. Several studies have examined the effects of soil use change on the abundance, taxonomic richness, and frequency of trophic groups [1, 23–25], but changes in FMT have been little explored [21]. According to feeding guilds, the main functional groups recognized in termites are xylophagous, humivorous, litter feeders, and intermediate [26].

Functional morphological trait studies facilitate the understanding of organisms’ responses to land use change and may generate reference predictions for ecosystem services [19]. In afforestation with pines in the Colombian East Plains, the abundance of the xylophagous species *Heterotermes tenuis* (Hagen, 1858) increased in comparison to the previous use of soil and the nearby gallery forest, in part attributed to the more significant amount of food available in the monocultures [6, 27]. However, the functional morphological responses that allow this species to adapt to changing environmental conditions are unknown.

In termites, the workers are responsible for foraging for food and building the nest. Therefore, the modifications related to trophic traits may impact this caste directly, so the worker caste has a more developed jaw than the other castes [28]. Likewise, the soldiers and nymphs cannot feed themselves, feeding from material regurgitated by the workers, and therefore, the workers provide the fundamental energy resource for the colony [26].

However, *Heterotermes tenuis* soldiers perform defensive and exploratory functions [29]. Therefore, changes related to the quantity and quality of available food will directly impact the FMT of termites. Likewise, FMT may express the caste of soldiers in response to predators, especially ants [28]. Given the transformations in the quantity and type of food under forest monoculture conditions, compared to pastures or gallery forests, changes are expected in the body size and the mouthparts of the workers of *H. tenuis,* while at the same time, aspects related to defense (for example, in the face of changes in the abundance of predators) will be reflected in the body size of the soldiers.

Here, we compared the size of various morphological and functional traits of worker and soldier castes at different ages of *Pinus caribaea* plantation (Morelet, 1851) and gallery forest relicts among different land uses in afforested areas of the East Plains in the Colombian Orinoquia. After estimating the intraspecific variation in those traits, we compared the FMT of foraging, feeding, and defense in the species *H. tenuis*. We expect to contribute to the functional perspective of this species' ecology and better understand the mechanisms of adaptive response to the alteration of the food source. This study will also contribute as the basis for predicting changes in ecosystem services derived from the activity of these insects, such as the rate of wood decomposition, nutrient regulation, and soil formation.

## Materials and methods

### Study area

**Fig. 1:**
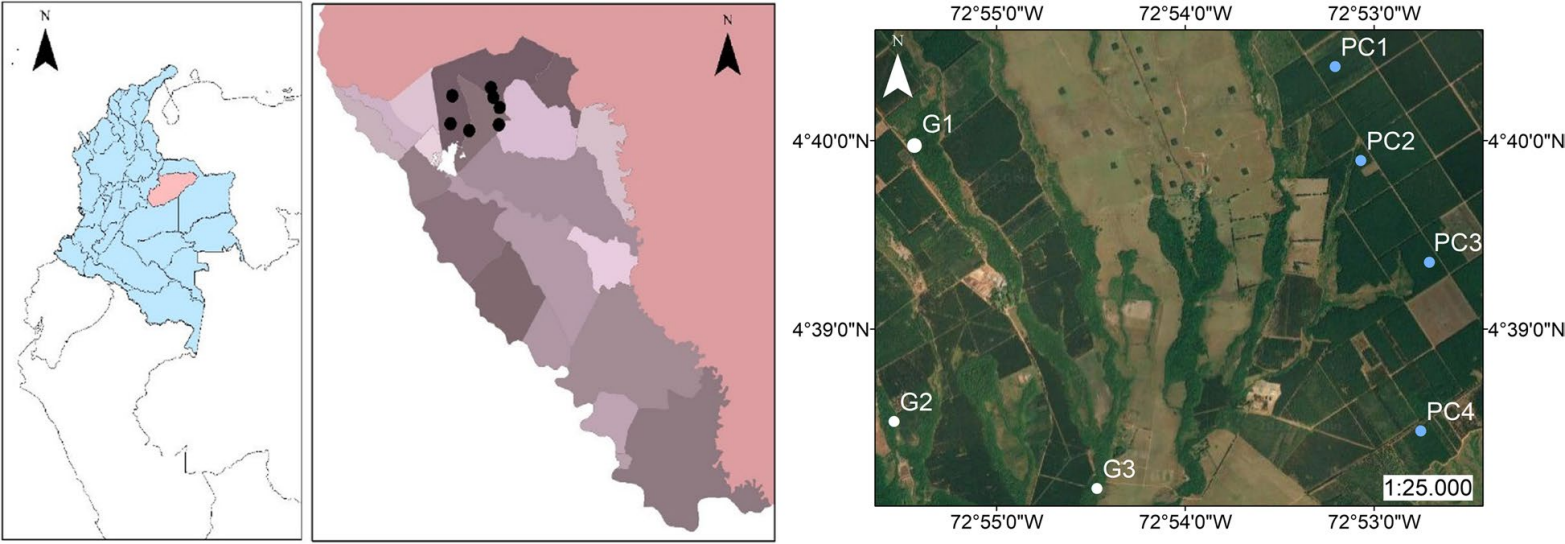
Location of the study area in Villanueva, Casanare, Colombia. The general geographical location of the sampling area. South America layer obtained from http://www.efrainmaps.es. Carlos Efraín Porto Tapiquén. Geografía, SIG y Cartografía Digital. Valencia, España, 2020 and Casanare layer from DANE Geoportal https://geoportal.dane.gov.co/geovisores/territorio/nivel-de-referencia-de-veredas/ (**A**). Sampling plot locations drawn on Google Earth images obtained using SAS Planet software http://www.sasgis.org/download/version May 19, 2023. White dots depict gallery forests, and blue dots pine plantations. PC1: 1–2 years old, PC2: 6–7 years old, PC3: 7–8 years old, PC4: 12–23 years old, G1 to G3: Gallery Forest (**B**)

Specimens of *H. tenuis* measured in this study were collected from *P. caribaea* plantations (area of 1,500 hectares) and relictual gallery forests in 2015 on the San Pedro plateau, Villanueva, Casanare, Colombia (4° 36′ 0″ N, 72° 55′ 1″ W) at 358 m altitude [6, 27], as shown in Fig. 1. The collection locality has a monomodal climatic regime, with an average annual temperature of 25.7 °C and precipitation of 2911 mm. The rainy period occurs from April to November, and the dry period occurs from December to March. The San Pedro plateau comprises a high terrace of sandy to sandy-clay soils of a quartzous nature and low fertility, belonging to the entisol and inceptisol orders [30].

### *Pinus caribaea* plantations and relicts of gallery forests

At the time of the collections, the *P. caribaea* plantations had plots of different ages. Collections were performed on plantations with ages of 1 to 2 years, 6 to 7 years, 7 to 8 years, and 19 to 23 years. On the other hand, the relicts of gallery forests surround the plantation plots [27], and their plant diversity and structure were previously studied [31]. The size of the trees in the plantation plots and gallery forest relicts studied, as well as soil pH, soil carbon content, percentage of canopy cover, and silvicultural management, are summarized in Table 1. The size of the trees in the plantation plots and gallery forest relicts studied, as well as soil pH, soil carbon content, canopy cover, and silvicultural management from [6, 27], are shown in Table 2.

**Table 1:** Characterization of the four ages of *Pinus caribaea* and gallery forest relicts, modified from [6] and [27]

Land use	Silvicultural management	DBH (cm)	Height (m)	pH	Organic carbon (%)	Canopy cover (%)
*Pinus caribaea* 1–2-year-old	Without thinning	7,2 ± 0,9	4,2 ± 0,7	4,4	1,3	25,4 ± 6,2
*Pinus caribaea* 6–7-year-old	Without thinning, pruning	18,7 ± 2,8	13,5 ± 2,7	4,3	1,1	28,3 ± 3,5
*Pinus caribaea* 7–8-year-old	With thinning, pruning	21,7 ± 5,0	14,8 ± 3,3	4,4	1,4	29,0 ± 2,7
*Pinus caribaea* 19–23-year-old	With thinning, pruning	24,5 ± 16,5	20,2 ± 10	4,1	1,2	31,6 ± 2,8
		11,7 ± 6,3	11,1 ± 2,5	4,3	4,3	84,2 ± 6,0
Gallery forest	No handling	13,6 ± 3,4	10,2 ± 4,7	4,5	1,2	84,0 ± 5,3
		24,2 ± 8,4	15,3 ± 3,3	4,4	3,0	86,3 ± 5,6

*DBH* Diameter at breast height

### Specimen selection

We used workers and soldiers of *H. tenuis* collected in 2015 as part of termite diversity studies in the area [6, 27]. Termites were collected following the standard transect method with modifications [6, 27, 32] and kept preserved in 80% ethanol in the Colección Entomológica Forestal CEFUD (RNC045) at the Universidad Distrital Francisco José de Caldas. We used measurements from seven workers and seven soldiers per colony of 15 colonies (105 workers and 105 soldiers) to establish possible changes in FMT due to land use. The number of individuals per sample follows [10]. The soldier caste of *H. tenuis* is dimorphic, but we measured only the minor soldiers due to their greater abundance in the samples. A maximum of one sample from each subtransect ensured a distance between 10 m (from an adjacent subtransect) to 50 m (if the sample came from the last subtransect) between samples, given that each transect had a 50 m length. According to the transect protocol, one sample per subtransect represents an independent colony.

### Measurement of functional morphological traits of *Heterotermes tenuis*

**Fig. 2:**
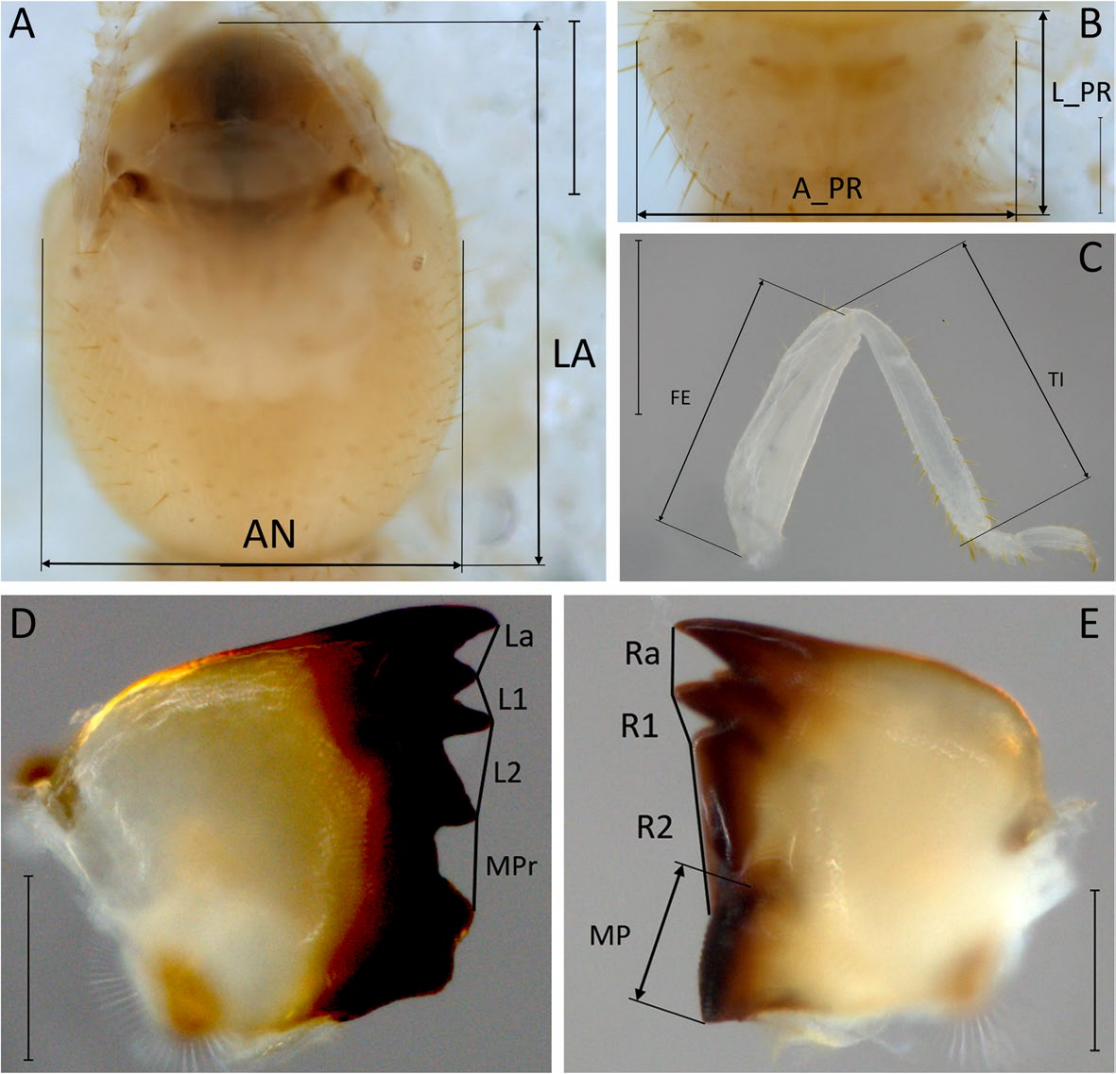
Worker dorsal view of the head at 50x (**A**), pronotum (**B**), prothoracic leg (**C**), and left mandible (**D**) and right mandible (**E**) at 60 × of a *Heterotermes tenuis* worker. Scale bars are 0,2 mm except for **A** and **C**, where it is 0,5 mm

**Fig. 3:**
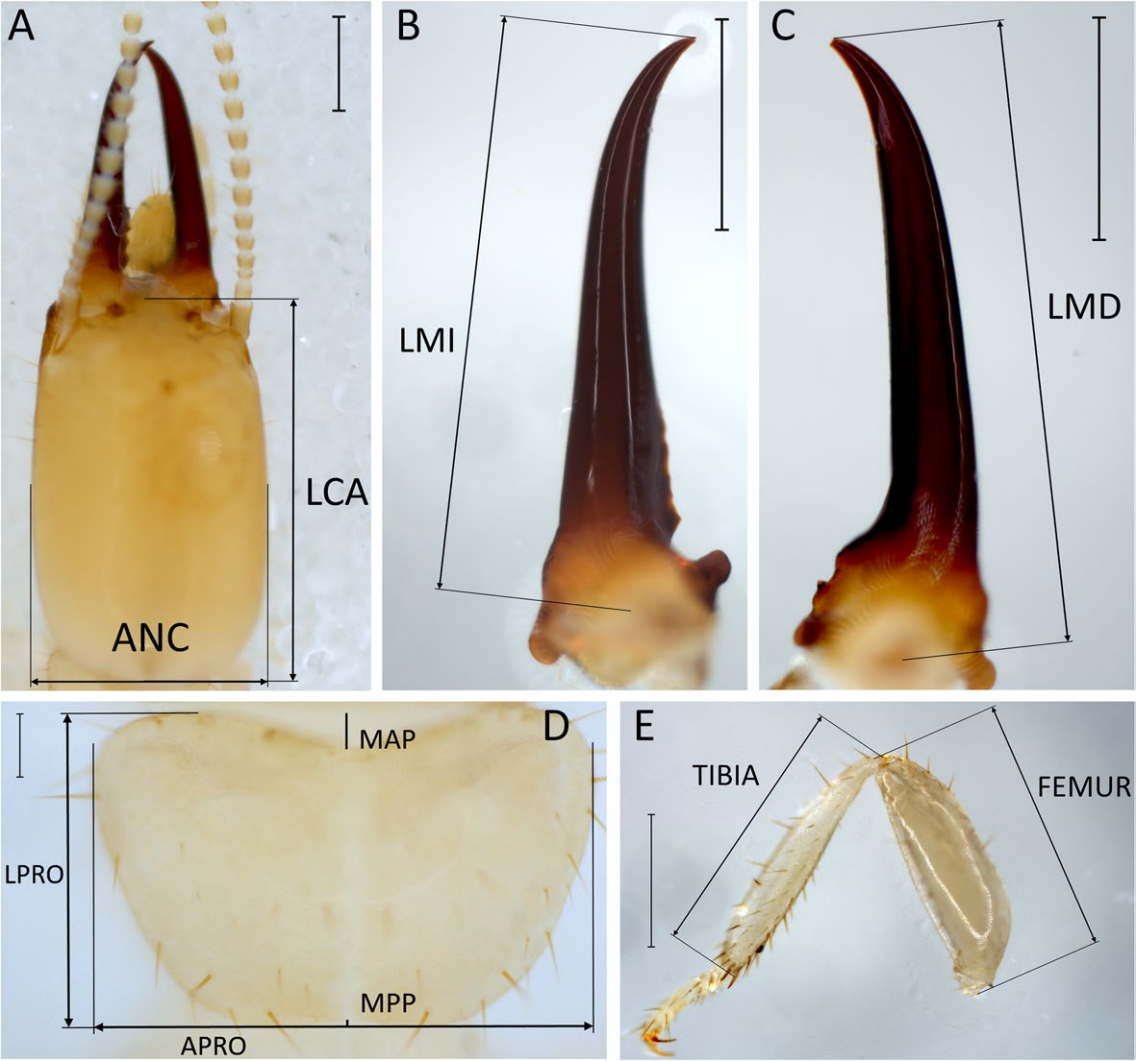
Minor soldier Dorsal view of the head at 50 × head (**A**), left mandible (**B**) and right mandible (**C**) at 60x, pronotum (**D**) and prothoracic leg (**E**) at 50 × of a *Heterotermes tenuis* minor soldier. Scale bars are 0.5 mm except for **D**, where it is 0.2 mm

Traits related to foraging in workers and defense activity in soldiers [21] included measurements on the head, mouthparts, and legs according to standard definitions in termites [33]. Fourteen morphological features of the thorax and head of the workers were included (Fig. 2): the maximum width (A_PR) and length (L_PR) of the pronotum, length of the tibia (TI) and femur (FE) of the prothoracic right leg, the maximum width (AN) and length (LA) of the head and the following characteristics of the mouthparts taken in the left mandible: the distances between the first apical tooth and the first marginal tooth (La), the first and second marginal teeth (L1), the second and third marginal teeth (L2), and the third marginal tooth and the molar prominence (MPr). In the right mandible, the distances between the first apical tooth and the first marginal tooth (Ra), the first and second marginal tooth (R1), the second marginal tooth and the molar plate (R2), and the second marginal tooth and the molar plate (R2) and molar plate extension (MP) are shown in Fig. 2. On the other hand, the FMT measured for the soldier caste included (Fig. 3): left (LMI) and right (LMD) mandible length, width, and the maximum length of the head (ANC and LCA, respectively), length and maximum width of the pronotum (LPRO and APR, respectively), anterior pronotal notch depth (MAP), posterior pronotal notch depth (MPP), and prothoracic leg tibia (TIBIA) and femur length (FEMUR).

Additionally, we used the following morphological indexes calculated according to [33] to estimate proportions between the RFM of both breeds.

Worker caste

- W1: Maximum head width/cephalic capsule length: cephalic capsule shape
- W2: Maximum length pronotum/maximum width pronotum: Shape of the pronotum
- W3: Maximum pronotum width/maximum head width: Head-pronotum ratio

Caste soldier

- S1: Maximum width of the head/length of the head to the lateral line of the mandibles: cephalic capsule shape
- S2I: Maximum left mandible length/maximum head width: Proportion of left mandible and head
- S2D: Maximum length of the right mandible/maximum width of the head: Proportion of right mandible and head
- S3: Maximum length of the pronotum/maximum width of the pronotum: Shape of the pronotum

We measured the FMT from high-resolution photographs obtained with an Axiocam 506 camera attached to a Carl Zeiss Discovery V8 stereomicroscope using the photography and measurement software ZEN (version 2.6).

### Analysis of data

#### Selection of functional morphological traits with less intraspecific variation

Principal component analysis (PCA) used land use as the explanatory variable to explore the FMT grouping patterns and identify those with the most significant contribution to the observed variance. For this grouping, the analysis categorized termite samples into five land use subgroups: 1–2 years, 6–7 years, 8–9 years, 19–23 years, and gallery forests. The coefficient of variation (CV) of each FMT was estimated to select those with lower intraspecific variation to compare plantations vs. gallery forests and among plantation ages. We used the raw measurements in mm to estimate the CV (%) using the formula: (Standard deviation/mean) *100. Additionally, we performed a univariate comparison among the size of the FMT values of both castes per colony and per land use using the nonparametric Kruskal‒Wallis (KW) test. Subsequently, using CV and KW, the FMT with the lowest intraspecific variation was obtained for both *H. tenuis* castes.

#### Effect of plantation age/land use on the size of functional morphological traits

To estimate changes in the FMT among different land uses, we calculated the community-weighted mean (CWM) index value corresponding to the weighted mean of each trait [34]. Additionally, we assumed that the colonies of each land use type were different species with the same abundance. We compared the plantation ages through a PERMANOVA with 999 permutations and a *p* value equal to or less than 0.05. Additionally, we compared the differences between the individuals of both colonies in the different land uses using nonmetric multidimensional scales (NMDS) with the Manhattan distance. For this purpose, we only considered the FMT with the lowest CV percentage. Then, to estimate the effect of land use and colonies on worker and soldier caste size, multinomial models were implemented for both cases. In addition, we included only the FMT with the lowest CV for all models for debugging. For each model, the following variables were included: mean diameter at breast height of trees in the transect (DBH), mean tree height, soil organic carbon, soil pH, and percentage canopy cover. We included land use as a factor for the first model, while the colony was the factor for the second model. In each model, the Akaike value and three pseudo R2 values (CoxSnel, Nagelkerke, and McFadden) were estimated to estimate which model presented better predictive quality and better explained the variation found in both castes. We used the R-Project V 1.4.1717 packages to perform all the analyses ([35], Appendix).

## Results

### Functional morphological traits with less intraspecific variation

**Fig. 4:**
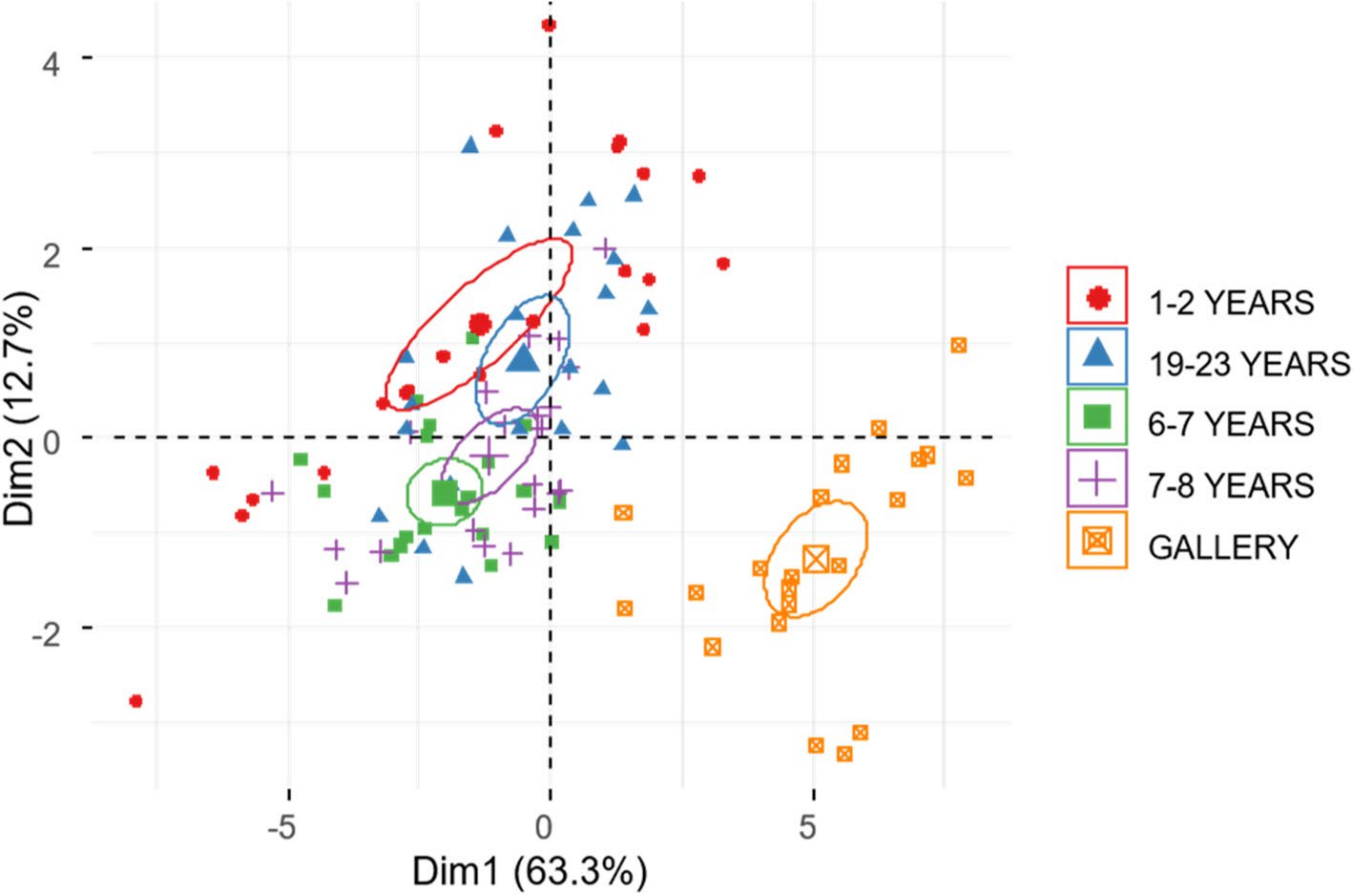
Morphological and functional traits of *Heterotermes tenuis* workers. Biplot of the type of land use as a descriptive variable. The ellipses represent groupings of individuals based on principal components

Workers of *H. tenuis* from plantations had smaller FMTs than workers from gallery forests (Fig. 4). The FMT of the first two axes of the PCA explained 83.3% of the observed variation. The first axis differentiated the gallery forest workers from the pine plantations, and the second axis separated the individuals from the intermediate and the younger and more mature plantations (Fig. 4).

**Fig. 5:**
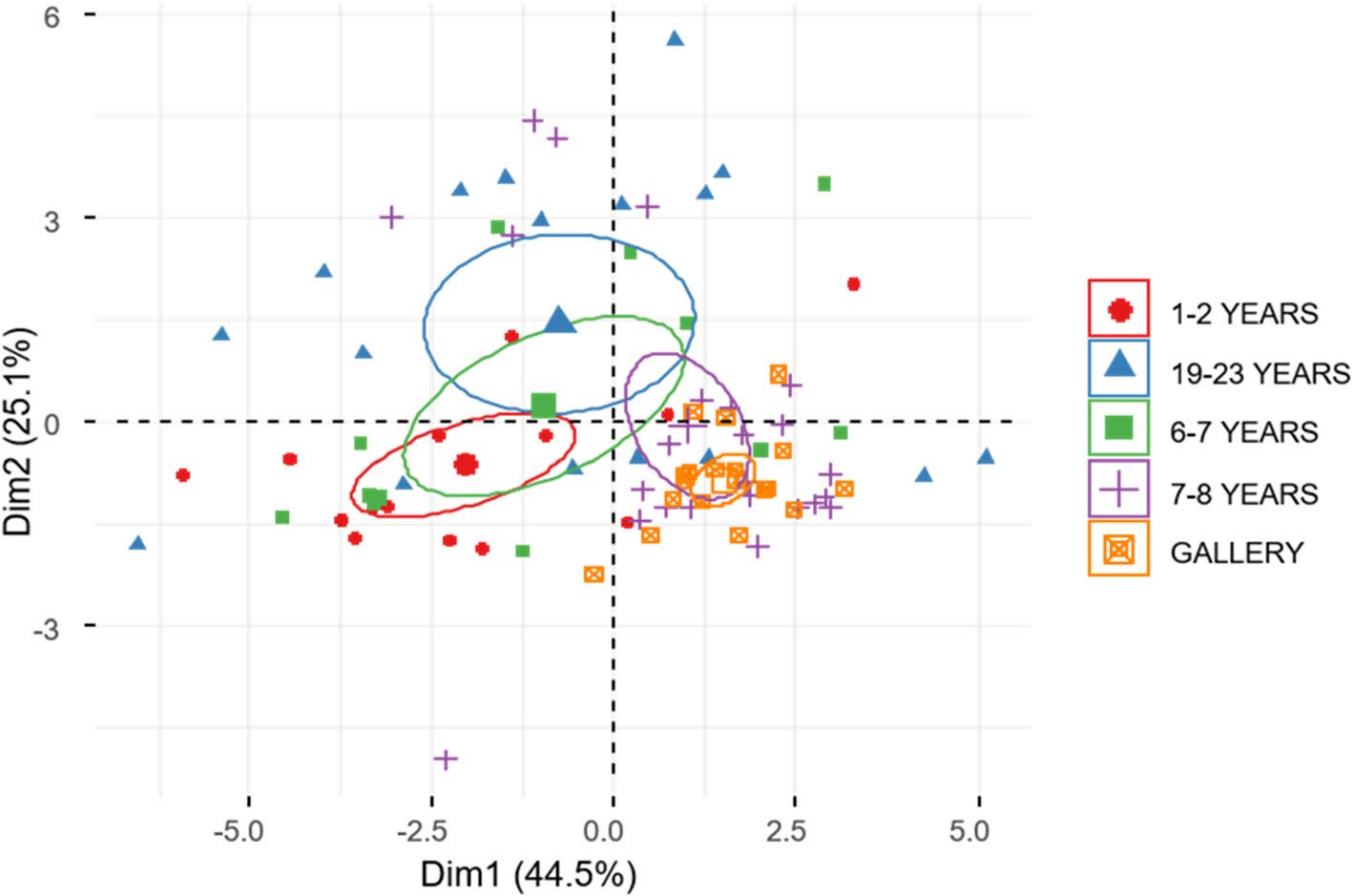
Morphological traits of the soldier caste *Heterotermes tenuis.* The biplot of the principal component analysis uses the land use type as a descriptive variable. The ellipses represent groupings of individuals based on principal components

Soldiers of *H. tenuis*, on the other hand, were partially separated based on the size of the FMT (Fig. 5). The PCA explained 71.6% of the observed variation, but in this case, the individuals from the gallery forests and the eight-year-old plantations were concentrated among the largest individuals.

The intraspecific variation in the FMT of *H. tenuis* ranged from 0.15% to 50.5%. In the worker caste, the FMT presented a CV between 0.15% and 18.5% at the colony and land use levels (Table 2). Head length had the highest CV, while mandibular trait L1 had the lowest value. In the soldier caste, the CV ranged from 0.14% to 50.5% (Table 2), with the posterior notch of the pronotum and the length of the tibia being the traits with the highest and lower CVs, respectively. The FMTs with lower CVs in the worker caste were L1, L2, MPr, Ra, R1, R2, MP, and the index W2 (Table 2; df:2, *p* value: 0.05), which are related to the worker's feeding activity. In the case of the soldier caste, the FMTs with the slightest variation were head width and length, pronotum width, maximum length of the mandibles, length of the tibia, Index S2I and Index S3 (Table 3), which are related to colony defense (df:2, *p* value: 0.05).

**Table 2:** Coefficient of variation (CV) expressed in % of the functional morphological traits of the worker caste of *Heterotermes tenuis* in three colonies of each land use type

COLONY	L1	La	L2	MPr	Ra	R1	R2	MP	W2	W3	W1	Pronotum length	Femur	Head width	Pronotum width	Tibia	Head width
1.1	0,48	0,49^*^	0,68^*^	0,63^*^	0,73^*^	0,74	1,68^*^	1,45	2,61	5,14^*^	4,88^*^	4,14^*^	4,41^*^	5,07^*^	6,08^*^	6,70^*^	8,56^*^
1.2	0,54	0,64^*^	0,67^*^	0,62^*^	0,95^*^	0,51	1,18^*^	1,09	3,14	3,29^*^	3,28^*^	3,31^*^	2,52^*^	2,79^*^	3,24^*^	8,23^*^	4,83^*^
1.3	0,44	0,49^*^	0,76^*^	0,82^*^	0,74^*^	0,29	1,06^*^	1,62	3,65	3,29^*^	3,63^*^	2,83^*^	4,67^*^	3,80^*^	4,01^*^	4,33^*^	6,29^*^
2.1	0,26	0,28	0,22	0,44	0,33	0,28	0,52^*^	0,72^*^	1,49	2,22^*^	4,89	1,67	2,68^*^	1,25^*^	2,09^*^	4,18^*^	6,13
2.2	0,24	0,32	0,44	0,17	0,35	0,45	0,69^*^	1,19^*^	5,40	2,80^*^	3,82	2,24	3,21^*^	3,29^*^	4,27^*^	6,27^*^	7,07
2.3	0,45	0,51	0,65	0,56	0,49	0,48	1,52^*^	1,68^*^	3,14	2,15^*^	6,71	1,89	3,60^*^	2,28^*^	1,34^*^	2,90^*^	7,43
3.1	**0,15**	0,33	0,26^*^	0,33^*^	0,38^*^	0,40	0,94	0,83	5,64	1,33	2,02	4,18	2,11^*^	1,89^*^	1,18^*^	1,59	3,00^*^
3.2	0,51	0,71	0,63^*^	0,65^*^	0,64^*^	0,71	1,60	1,66	3,67	3,05	2,74	4,43	4,33^*^	4,98^*^	5,51^*^	6,57	9,55^*^
3.3	0,23	0,27	0,38^*^	0,32^*^	0,34^*^	0,64	1,07	1,52	4,10	2,75	4,38	2,41	5,77^*^	2,67^*^	2,29^*^	5,74	5,27^*^
4.1	0,35	0,61	0,48^*^	0,62	0,70	0,43	1,18^*^	0,72^*^	2,97	4,37	6,81	3,39	4,21	2,94	4,32^*^	3,50	**18,52**
4.2	0,32	0,23	0,32^*^	0,64	0,29	0,24	0,89^*^	0,98^*^	7,55	3,02	2,79	7,43	7,10	5,52	5,28^*^	5,79	8,65
4.3	0,30	0,27	0,49^*^	0,71	0,44	0,22	0,86^*^	1,34^*^	3,10	3,00	3,11	2,56	3,45	2,89	3,32^*^	3,49	5,87
5.1	0,41^*^	0,55	0,66	0,49	0,50	0,45	1,36	1,03^*^	1,70	1,68	5,05^*^	2,47	4,83^*^	5,15^*^	3,89^*^	7,54^*^	10,59
5.2	0,47^*^	0,33	0,51	0,71	0,55	0,32	1,44	0,94^*^	1,35	5,65	4,63^*^	3,96	3,13^*^	2,05^*^	6,89^*^	4,09^*^	10,48
5.3	0,48^*^	0,56	0,56	0,55	0,62	0,62	0,49	0,86^*^	5,80	3,15	3,06^*^	5,35	3,90^*^	4,84^*^	2,69^*^	5,41^*^	6,48

Colonies are organized by planting age: 1.1 to 1.3 (1–2 years), 2.1–2.3 (6–7 years), 3.1–3.3 (7–8 years), 4.1–4.3 (19–23 years), and 5.1- 5.3 (gallery forests). La-Mpr (Left Mandible): distance between the first apical tooth and the first marginal tooth (La), the distance between the first and second marginal tooth (L1), the distance between the second and third marginal tooth (L2), and the distance between the third marginal tooth and the molar prominence (MPr). Ra-MP (Right Mandible): distance between the first apical tooth and the first marginal tooth (Ra), KW distance between the first and second marginal teeth (R1), distance between the marginal second tooth and molar plate (R2), and molar plate extension (MP). W1-W3: Indexes of workers ^*^KW traits that differ statistically significantly from colonies of the same plantation age/gallery forest with a *p* value of 0.05. Df:2 N:7 individuals measured per colony. Extreme values of percent CV are in bold font

**Table 3:** Coefficient of variation (CV) expressed in % of the functional morphological traits of the soldier caste of *Heterotermes tenuis* in three colonies of each land use type

COLONY	Tibia length	S3	Lenght left mandible	S1	Mandible length Rig	S21	Head width	Pronotum width	Head length	S2D	Femur length	Pronotum length	Anterior notch pronotum	Posterior notch pronotum
1.1	5,15	8,71	10,49	6,97	12,7	6,37	8,97	9,91	14,71	8,07	9,36	18,74	17,82	41,98
1.2	7,49	3,77	4,94	2,71	6,06	1,32	6,27	3,84	3,53	1,24	5,39	6,72	11,82	24,25
1.3	14,99	7,58	13,15	5,29	13,87	6,21	9,68	11,62	11,91	5,02	15,37	15,19	13,12	24,65
2.1	13,84	4,52	12,13	6,13	13,94	8,73	5,03	9,86	10,45	9,86	15,52	13,50	36,41	35,85
2.2	8,92	11,36	6,63	6,64	15,57	11,33	14,42	11,86	19,14	22,45	9,18	22,65	25,21	37,88
2.3	**0,14**	5,10	2,39	14,00	1,11	10,68	13,06	9,2	0,95	14,16	9,58	14,27	14,54	26,94
3.1	9,27	1,47	5,42	4,12	5,13	13,60	9,52	8,71	9,27	13,48	2,80*	9,43	16,62	**50,56**
3.2	8,80	6,33	10,02	3,29	11,55	19,47	14,28	12,3	13,27	23,86	10,28*	15,81	18,40	37,55
3.3	4,41	4,31	3,21	10,05	2,33	3,51	3,4	1,89	8,76	4,63	1,16*	4,04	16,06	31,81
4.1	12,48*	12,19	11,16	8,38*	11,65	13,46	11,12	13,81	14,62	13,51	11,53*	20,63	32,47	27,72
4.2	4,57*	2,32	1,88	5,39*	14,55	2,51	0,73	1,06	5,65	14,69	5,71*	2,69	38,92	14,29
4.3	6,19*	5,64	5,75	44,33*	5,05	14,29	17,4	17,88	20,46	15,36	5,25*	22,07	20,16	47,69
5.1	2,56	3,67*	4,65	4,05*	2,81	5,83	3,09	3,64	3,93	5,16	4,20*	1,84	8,96	32,67
5.2	5,01	3,33*	4,78	3,00*	4,72	2,98	4,13	7,64	5,27	2,41	4,47*	5,02	14,46	39,73
5.3	2,91	3,52*	1,54	3,48*	1,01	4,19	3,85	4,09	4,08	3,11	3,23*	3,08	11,70	36,12

Colonies are organized by planting age: 1.1 to 1.3 (1–2 years), 2.1–2.3 (6–7 years), 3.1–3.3 (7–8 years), 4.1–4.3 (19–23 years), and 5.1- 5.3 (gallery forests). S1-S3: Indexes of minor soldiers’ ^*^KW traits present statistically significant differences between colonies of the same age of plantation/gallery forest with *a p* value of 0.05. Df:2 N:7 individuals measured per colony. Extreme values of percent CV are in bold font

### Effect of land use and plantation age on the size of functional morphological traits

#### Workers

For the worker caste, the specific traits of the left (La, L1, L2, Mpr) and the right mandible (Ra, R1, R2, MP) increased in the mature plantations (19–23 years) and the gallery forest relicts, while W2 was higher in the younger and more mature plantations (1–2 years, 19–23 years; Table 4). The average size of the FMT selected from the workers of pine plantations was minor compared to that of gallery forests (PERMANOVA (df:4, p value: 0.05). Likewise, there were differences between the average sizes of the individuals of the plantations of intermediate ages (6–8 years) and the mature plantations (19–23 years; Additional file 1).

**Table 4:** CWM index of the worker and soldier caste of *Heterotermes tenuis* in the land use

**Caste**	**Land use**			**Functional morphological features**						
		**La**	**L1**	**L2**	**MPr**	**Ra**	**R1**	**R2**	**MP**	**W2**
**Workers**	*Pinus caribaea* 1–2-year-old	0,05	0,04	0,08	0,08	0,06	0,05	0,16	0,14	0,57
	*Pinus caribaea* 6–7-year-old	0,05	0,04	0,08	0,08	0,06	0,05	0,16	0,14	0,54
	*Pinus caribaea* 7–8-year-old	0,05	0,04	0,08	0,08	0,06	0,05	0,16	0,14	0,53
	*Pinus caribaea* 19–23-year-old	0,05	0,05	0,09	0,08	0,07	0,05	0,17	0,14	0,58
	Gallery forest	0,06	0,06	0,1	0,1	0,08	0,06	0,21	0,18	0,52
		**ANC**	**LCA**	**LMI**	**LMD**	**APR**	**TIBIA**	**S2I**	**S3**	
**Minor Soldiers**	*Pinus caribaea* 1–2-year-old	0,969	1,5	1,17	1,19	0,7	0,81	1,21	0,63	
	*Pinus caribaea* 6–7-year-old	1,01	1,55	1,25	1,26	0,71	0,89	1,25	0,62	
	*Pinus caribaea* 7–8-year-old	1,12	1,65	1,33	1,35	0,78	0,92	1,2	0,65	
	*Pinus caribaea* 19–23-year-old	0,994	1,5	1,33	1,35	0,71	0,91	1,36	0,64	
	Gallery forest	1,17	1,71	1,32	1,33	0,8	0,94	1,12	0,65	

*La* distance between the first apical tooth and the first marginal tooth, *L1* distance between the first and second marginal teeth, *L2* distance between the second and third marginal teeth, *MPR* Molar prominence, *Ra* distance between the first apical tooth and the first marginal tooth, *R1* distance between the first and second marginal teeth, *R2* distance between the second marginal tooth and the molar plate, *MP* Molar plate. *W2* Shape of the pronotum of the worker. *ANC* width of the head, *LCA* Length of the head, *LMI* maximum left mandible length, *LMD* maximum right mandible length, *APR* pronotum width, *S2I* proportion of left mandible and head, *S3* shape of the pronotum of the soldier

**Fig. 6:**
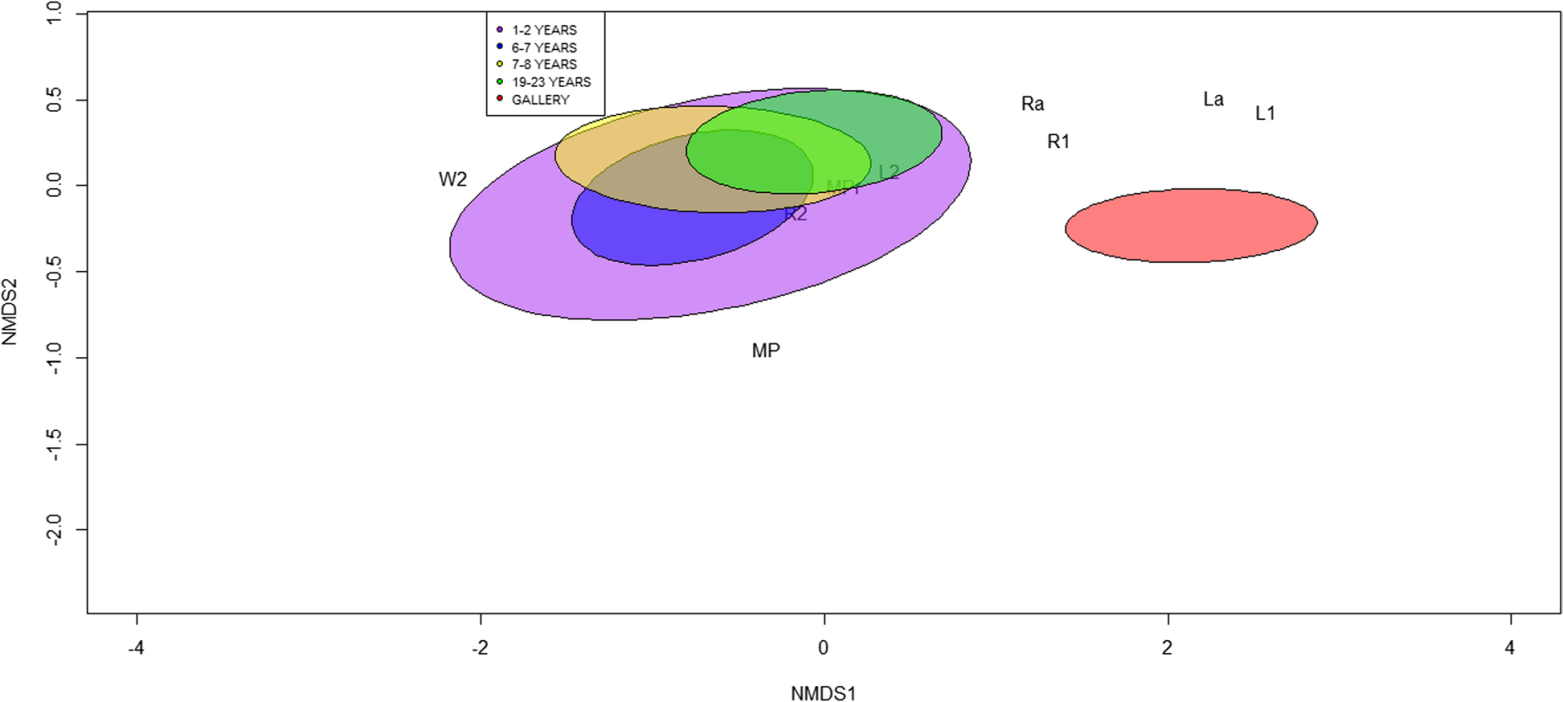
Nonmetric multidimensional ordination (NMDS) of the size of the FMT of the worker caste of *Heterotermes tenuis* in pine plantations of different ages and gallery forests. La: distance between the first apical tooth and the first marginal tooth, L1: distance between the first and second marginal tooth, L2: distance between the second and third marginal tooth, MPR: molar prominence, Ra = distance between the first apical tooth and the first marginal tooth, R1: distance between the first and second marginal teeth, R2 distance between the second marginal tooth and the molar plate, MP: molar plate. W2: Shape of the pronotum

The ordination analysis supported a strong correlation between the size of the FMT and the land use. Two subgroups within the worker caste were observed (Fig. 6). The first group included colonies of pine plantations, while the second group included colonies of relicts of gallery forest. The FMT of the workers in the first group was smaller than that of the second group. The prominence and molar plate, R2, L2, and pronotum shape defined group one, while group two was more related to the traits Ra, La, R1, and L1 (Fig. 6).

#### Minor soldiers

**Fig. 7:**
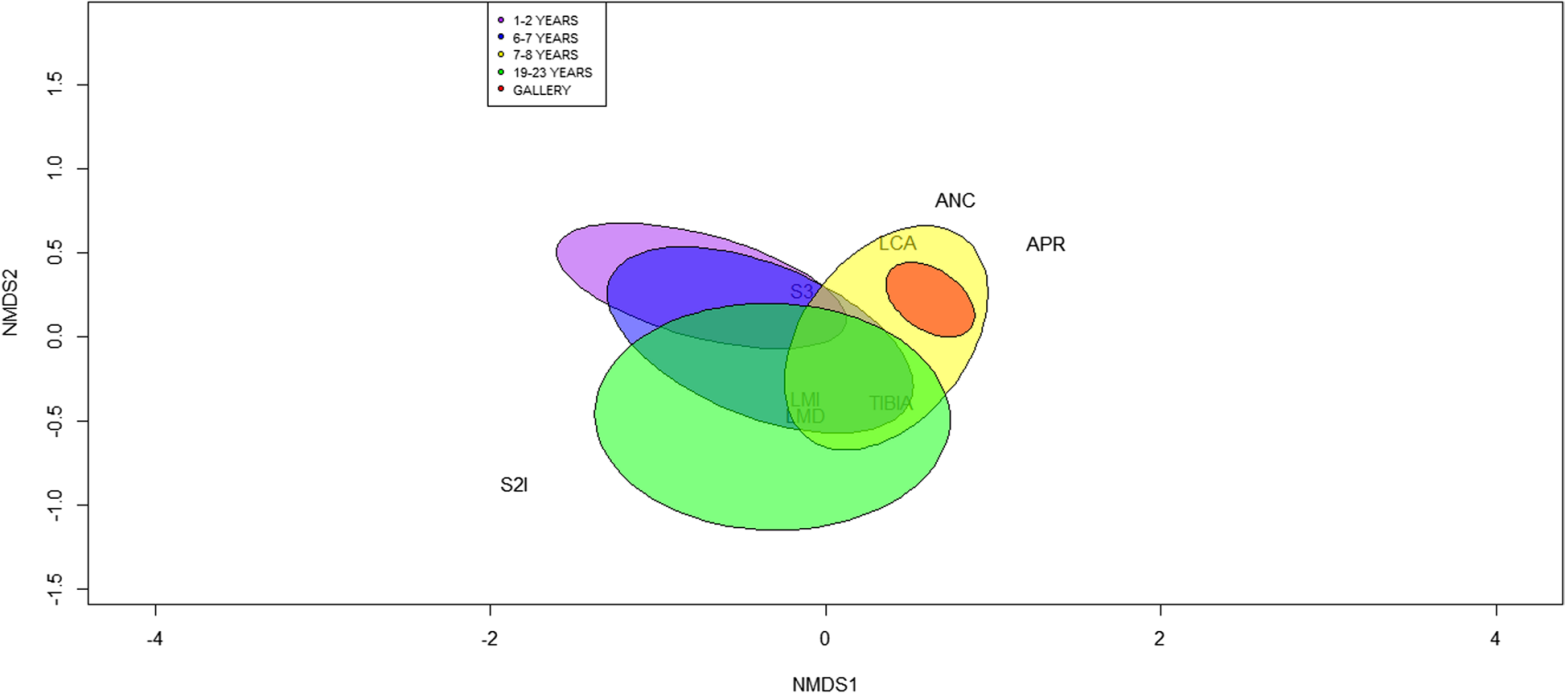
Nonmetric multidimensional scaling (NMDS) ordination of the soldier caste of *Heterotermes tenuis* in pine plantations of different ages and gallery forests. ANC: width of the head, LCA: length of the head, LMI: maximum left mandible length, LMD: maximum right mandible length, APR: pronotum width, S2I: proportion of left mandible and head, S3: shape of the pronotum

For the soldier caste, traits related to head size, pronotum, length of both mandibles and tibia, and indexes 2I and 3 increased in size in gallery forests and plantations aged 7 to 8 years (Table 4). For the FMT and the CWM, values behaved differently than the worker caste, forming two subgroups. Individuals from 6- to 8-year-old plantations and gallery forests presented larger FMTs than the rest of the plantation ages for most CWM values. The PERMANOVA results (df:4, *p* value: 0.05) show differences between the youngest plantations (1–2 years) and the intermediate plantations (7–8 years). Likewise, there were differences between plantations of 1–2 years, 6–7 years, and 19–23 years with the gallery forests (Additional file 1). In addition, the ordination analysis showed a strong correlation between the FMT of the soldier and the type of land use (Fig. 7), forming two groups. The first group corresponded to the colonies of the youngest pine plantation ages (1–2 years; 6–7 years) and more mature (19 to 23 years). Their size was significantly smaller than that of the soldiers of group two, which corresponded to the gallery forests. Group one was defined by the length of both mandibles, the length of the tibia, and Index 3, while group two was related to the width and length of the head and the length of the pronotum (Fig. 7).

#### Multinomial models

The multinomial models of the worker and minor soldier caste helped estimate a possible homogenization of FMT. The Akaike predictive quality value was lower when land use was a factor, in contrast to the value obtained when the colony was a factor. Likewise, the models explained a large part of the variation observed, showing values of the three pseudo R^2^ equal to or close to 1, and therefore are considered robust. The models obtained from the soldier caste were slightly less robust than those obtained from the worker caste (Table 5).

**Table 5:** Multinomial models of the worker and soldier caste of *Heterotermes tenuis* using colonies and land use as factors

**Caste**	**Response**	**AIC**	**Pseudo R2**			**Predictor**
Worker	Land use	112	0,96	1	0,99	FMT with lower CV, DAP, C organic, average height, % canopy cover, pH
	Colony	392	0,99	1	0,99	
Minor Soldier	Land use	112	0,96	0,99	0,99	FMT with lower CV, DAP, C organic, average height, % canopy cover, pH
	Colony	392	0,95	0,99	0,99	

*CV* Coefficient of variation

## Discussion

The size of the FMT of the workers and minor soldiers of *H. tenuis* was influenced by the land use and, to a lesser extent, by the ages of the pine plantations. Likewise, the IV of 16 traits out of the 38 studied presented lower CV values in both plantations and gallery forests, minimizing the effect of intraspecific variability on land use comparisons.

### Variation in functional morphological traits of *Heterotermes tenuis* workers and soldiers

The more variable FMTs in the worker caste of *H. tenuis* were those related to general body size (head, thorax, and extremities), that is, to the food search. In contrast, the less variable FMTs were the characteristics of the mouthparts, which affected food foraging. Significant intraspecific variation related to the active search for food between and within populations is already known from social insects [36, 37]. Thus, highly variable traits can improve colony fitness by extending the range of behavior (for example, foraging) and allowing a better and faster response to changes [36]. In addition, the time invested in searching for food varies due to resource availability, which in the case of the Rhinotermitidae family corresponds to decomposing wood [5]. Additionally, the fungal decomposition of wood is associated with a higher feeding rate of workers due to increased nitrogen [38]. Therefore, it is possible that the more active *H. tenuis* workers have larger body sizes, and consequently, the variation in certain traits increases as an adaptive mechanism.

The FMT with the most significant variation in the soldier caste was the general size of the insect body (thorax and extremities), the shape of the head, and the size ratio of the head/right jaw, while the size of the head, the size of the pronotum and the length of both mandibles were the less variable characteristics. Similar observations occur in the xylophagous termite *Cryptotermes secundus* (Hill, 1925), where the size of the features related to mechanical defense was more stable than the nondefensive morphological features [39]. Another explanation is related to the fact that the soldier caste of Rhinotermitidae initiates the exploration of the resource as a possible response to reduce worker predation [29, 40], and between colonies, the foraging time and the time of exposure of the workers to predation is variable [36]. Since in the present study, we measured the minor soldier, these individuals may be more related to the scouting activity in the exploration of food by the workers than in the function of defense [38], and therefore there could be a more significant intraspecific variation related to the movement characters for exploration than defensive ones. Therefore, we suggest developing future research on older and younger soldiers' functional and behavioral variation.

The FMT of the soldier caste of *H. tenuis* was more variable than that of the worker caste. In termites, the difference in the CV of the size of morphological traits between castes responds to several origins [28] and high developmental plasticity with complex epigenetic factors [41–43]. First, the soldier caste of termites may originate from all stages of workers and immature apterans, which can cause differences in final individual size polymorphisms in various species [44]. In addition, the size of the workers from which the soldiers originate in incipient colonies is smaller [43, 45], and therefore, their final size is expected to be partially dependent on the development pathway [46]. Therefore, younger plantation workers may produce smaller soldiers. Furthermore, polymorphism in the soldier caste of termites is related to polyethism (the division of labor) and has different patterns in the genus *Heterotermes* [47]. Likewise, intraspecific competition between colonies that share one or several substrates can be a stress factor influencing the size of some morphological traits in the individual development of soldiers [48].

Therefore, the traits that presented the lowest intraspecific CV were considered the most appropriate to estimate the land use effect on the species' functional response. Traits with lower CV minimize the possible overlapping or oversizing of the functional response due to high variation [10]. The traits considered most appropriate for comparison, including the worker caste, were the mandibular traits (La, L1, L2, MPr, Ra, R1, R2, MP) and W2 and the soldier caste: the width and length of the head, the width of pronotum, length of the tibia and both mandibles and indexes S2I and S3.

### Relationship of the size of morphological features with land use

*Heterotermes tenuis* workers from the gallery forests were larger than those from the pine plantations, regardless of the age of the plantation. This result coincides with the reduction in the FMT size of termites reported in *Hevea brasiliensis* monocultures compared to deforested natural forest areas and is attributed to the greater variety of food resources, microhabitats, and microclimates in the latter [21]. Nevertheless, the quality and quantity of food resources and environmental conditions must also be considered since the frequency of wood-eating termites increases with food availability in deforested areas [22].

The termite worker size is related to physiological factors, such as nutrition and energy expenditure, and mainly to the quantity and quality of food, although the results are contrasting. The larger size of worker termites may affect their ability to absorb nutrients [49] but at the same time may favor a lower rate of energy expenditure than small and medium-sized termites [49]. Additionally, larger termites may have a longer food retention time by having larger intestines, which is beneficial, considering that wood is a nutritionally poor substrate [49]. However, the largest workers of the Rhinotermitidae species, known as the Formosan subterranean termites (*Coptotermes formosanus* Shiraki, 1909), consume less wood and therefore suffer higher mortality than small workers, especially in declining colonies, which produce larger and less vigorous termites [50]. On the other hand, wood-eating termite workers can benefit from smaller sizes because the smaller mandibles can grind the food into finer pieces, improving the absorption of nutrients [51].

In the study area, the frequency of *H. tenuis* increases as *P. caribaea* ages [6] compared to the surrounding natural forests [27]. Additionally, in the study area, a positive response of the frequency of this species to the degree of decomposition and the size of the pieces of pine wood produced by pruning is known [6]. The frequency of subterranean xylophagous termites responds positively to the availability of wood resources they require for food and habitat. Moreover, Rhinotermitidae prefers to feed on soft woods such as *Pinus* [52] and can feed on needles of this genus [53]. The smaller mouthparts FMT of *H. tenuis* observed in the plantations may correspond to a response to the lower resistance of the pine wood. At the same time, in the gallery forests, the food consists of heterogeneity of woods [31] so that a smaller size of the mouthparts can be adequate and imply energy savings. Alternatively, the smaller size of the workers could be due to the nutritional supply of pine wood not allowing optimal body development. However, in this study, individual biomass was not compared between land uses [53].

On the other hand, the influence of interspecific competition among xylophagous species of termites on the size of individuals must be considered. Competition influences offspring size because competitive environments encourage the colony to invest in larger individuals but in smaller numbers [54]. Furthermore, competition is relevant in organisms that tend to saturate their environments [54], such as ants and termites. The workers of *H. tenuis* of the four plantation ages would have less interspecific competition compared to the workers of the relict gallery forests because the composition of xylophagous species was 24% in the pine plantations [3] and 42.7% in gallery forest relicts [5]. Therefore, it is possible that *H. tenuis* gallery forest workers increase the size of their FMTs and decrease their abundance, while plantations present smaller FMTs but a more significant number of individuals.

The size of the FMT of *H. tenuis* soldiers in this study differentiated for the workers since soldiers tend to be smaller in the youngest and most mature plantations compared to intermediate plantations and gallery forests. The variation in the size of the soldier caste of termites is mainly in response to their predators [28], and as also occurs in other social insects, traits associated with nest defense and locomotion increase proportionally to the abundance of insect predators because a larger size may be a better defense [55]. Likewise, ants are the most important predators of termites [56] and can reduce termite densities, even if they do not specialize in their predation [57]. Therefore, the difference observed in the size of some morphological characters of the minor soldier of *H. tenuis* may be due to the diversity and frequency of predators. However, we did not include this variable in the study.

Likewise, intraspecific competition may increase the variation between the different ages and land use for the soldier caste. For example, in the species *Neotermes chilensis*, it was found that individuals of the soldier caste, coming from colonies that shared the food substrate, increased the size of the head in comparison to individuals who did not share the substrate [48]. Increasing the size of the heads of the individuals of this caste increases the possibility of winning battles and monopolizing resources [48]. Therefore, intra- and interspecific competition may be a stress factor for the minor soldiers of *H. tenuis*. However, this should be analyzed cautiously since *N. chilensis* is a one-piece nest termite with habits that are different from those of *H. tenuis*.

On the other hand, incipient colonies of some termite species, having a low number of workers, produce smaller soldiers, while more mature colonies tend to produce larger soldiers [39, 58–60]; this would partly explain why individuals of the soldier caste tend to be larger in intermediate plantations and in gallery forests, where colonies may be more mature than in younger plantations. Another factor influencing the differences observed in the size of the functional traits of the workers and soldiers is the sex of the individuals, but we did not study that variable.

### Homogeneity of functional diversity

Intraspecific variation in *H. tenuis* FMT was more significant between colonies than among land use types. The multinomial models improved their predictive quality and the adjustment of the pseudo R2 when using the type of land use as a factor. Therefore, more significant variation is likely between colonies than land use. This variation between colonies may be due to the range of individual phenotypes in one colony differing from the range in another [37]. However, despite the high variation between colonies, its importance is relegated by the effect of land use on the insect's body. Therefore, *P. caribaea* plantations may act as a filter for the functional diversity of *H. tenuis* since no relevant differences between the plantation ages regarding FMT size, especially in the worker caste, were detected.

Homogenization consists of an increase in the similarity of the functional composition [18]. This homogenization can alter some ecosystem processes, such as waste decomposition and nutrient cycling [18], alter food webs [14], and generate losses in taxonomic diversity [16, 17]. Although the pine-planted areas do not correspond to transformed gallery forests, the results observed were similar to those reported for converting primary forests to rubber plantations [16, 21]. Rubber plantations were found to reduce the functional diversity of ants and termites due to a loss of microhabitats compared to primary forests. Primary forests have greater structural complexity, variety of resources, and microclimates, facilitating the coexistence of more functional features, while plantations limit specific traits. Additionally, functional diversity decreases with higher levels of disturbance [61, 62]; therefore, it is likely that pine plantations present more significant disturbance than the relicts of gallery forests, a decrease in the functional diversity of both castes. Therefore, pine plantations may restrict the functional variability of *H. tenuis* to specific traits, with a higher incidence in the worker caste.

## Conclusions

Land use affected the size of the functional morphological trait of *H. tenuis,* expressed in the smaller size of the workers in the plantations compared to the gallery forests. The observed size difference suggests an environmental filter effect of the plantations homogenizing the functional traits of the *H. tenuis* workers favoring the establishment of smaller functional traits.

Morphological traits of the worker and soldier castes related to the general size of the insect exhibited the highest percentages of coefficient of variation. Therefore, they are less valuable for evaluating the functional response of termites to changes in land use. To better understand wood decomposition processes in new forested areas, we recommend deepening studies referring to the effects on diversity and functional effects on soil macrofauna.

### Supplementary Information

**Additional file 1: Appendix 1.** Sampling scheme through the modified transect method of Jones et al., 2005 (Pinzón et al., 2017; Beltrán et al., 2018). **Appendix 2.** PERMANOVA of the worker caste of Heterotermes tenuis in four ages of Pinus caribaea plantation and gallery forest relicts. Df: 4, p value: 0.05, 999 permutations. **Appendix 3.** PERMANOVA of the soldier’s caste of Heterotermes tenuis in four ages of Pinus caribaea plantation and gallery forest relicts. Df: 4, p value: 0.05, 999 permutations. **Appendix 4.** R Studio scripts used for the different data analyses.

## Data Availability

We included the R Studio codes and PERMANOVA results as supplementary material. The database can be available by request to the corresponding author’s mail.

## Abbreviations

FMT: Functional morphological traits
*H. tenuis*: *Heterotermes tenuis*
*P. caribaea*: *Pinus caribaea*
*N. chilensis*: *Neotermes chilensis*
IV: Intraspecific variability
DBH: Diameter at breast height
CEFUD: Forest Entomological Collection of the Francisco José de Caldas District University

## Worker caste

La: Distance between the first apical tooth and the first marginal tooth
L1: Distance between the first apical tooth and the first marginal tooth
L2: Distance between the first and second tooth marginal
MPr: Distance between the third marginal tooth and the molar prominence
Ra: Distance between the first apical tooth and the first marginal tooth
R1: Distance between the first and second marginal tooth
R2: Distance between the second marginal tooth and the molar plate
MP: Molar plate extension
W1: Maximum head width/cephalic capsule length: cephalic capsule shape
W2: Maximum length pronotum/maximum width pronotum: Shape of the pronotum
W3: Maximum pronotum width/maximum head width: Head-pronotum ratio

## Soldier caste

LMI: Left mandible length
LMD: Right mandible length
ANC: Maximum width of the head
LCA: Maximum length of the head
LPRO: Maximum length of the pronotum
APR: Maximum width of the pronotum
MAP: Anterior pronotal notch
MPP: Posterior pronotal notch depth
S1: Maximum width of the head/length of the head to the lateral line of the mandibles
S2I: Maximum left mandible length/maximum head width
S2D: Maximum length of the right mandible/maximum width of the head
S3: Maximum length of the pronotum/maximum width of the pronotum
PCA: Principal Component Analysis
CV: Coefficient of Variation
KW: Kruskal‒Wallis
ANOVA: Analysis of variance
CWM: Community-weighted mean
NMDS: Nonmetric multidimensional scales
