# Supplementary material for: Ecology, behavior and bionomics: functional response of Heterotermes tenuis Hagen (Insecta: Blattaria: Isoptera: Rhinotermitidae) in forests of the Colombian Orinoquía
Source: BMC Zool. 2023 Oct 26;8:24. doi: 10.1186/s40850-023-00184-7 (PMC10601117; doi:10.1186/s40850-023-00184-7)
Supplement: Supplementary file 1 — Additional file 1: Appendix 1. Sampling scheme through the modified transect method of Jones et al., 2005 (Pinzón et al., 2017; Beltrán et al., 2018). Appendix 2. PERMANOVA of the worker caste of Heterotermes tenuis in four ages of Pinus caribaea plantation and gallery forest relicts. Df: 4, p value: 0.05, 999 permutations. Appendix 3. PERMANOVA of the soldier’s caste of Heterotermes tenuis in four ages of Pinus caribaea plantation and gallery forest relicts. Df: 4, p value: 0.05, 999 permutations. Appendix 4. R Studio scripts used for the different data analyses. [file 40850_2023_184_MOESM1_ESM.docx]

# Additional file 1

**Appendix 1**. Sampling scheme through the modified transect method of Jones et al., 2005 (Pinzón et al., 2017; Beltrán et al., 2018).


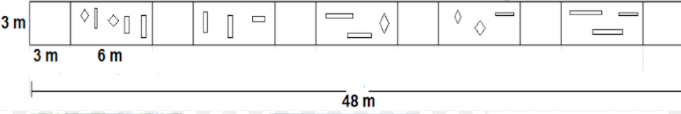


**Appendix 2**. PERMANOVA of the worker caste of *Heterotermes tenuis* in four ages of *Pinus caribaea* plantation and gallery forest relicts. Df: 4, *p* value: 0.05, 999 permutations

| **Contrast groups** | **Workers** | |
| --- | --- | --- |
|  | **F** | **P** |
| *Pinus caribaea* 1-2 years-Gallery Forest | 51,98 | 0,002 |
| *Pinus caribaea* 6-7 years- Gallery Forest | 68,1 | 0,002 |
| *Pinus caribaea* 7-8 years- *Pinus caribaea* 19-23 years | 59,59 | 0,002 |
| *Pinus caribaea* 19-23 years- Gallery Forest | 59,28 | 0,002 |

**Appendix 3**. PERMANOVA of the soldier's caste of *Heterotermes tenuis* in four ages of *Pinus caribaea* plantation and gallery forest relicts. Df: 4, *p* value: 0.05, 999 permutations

| **Contrast groups** | **Soldiers** | |
| --- | --- | --- |
|  | **F** | **P** |
| *Pinus caribaea* 1-2 years- *Pinus caribaea* 7-8 years | 11,7 | 0,002 |
| *Pinus caribaea* 1-2 years- Gallery Forest | 28,35 | 0,002 |
| *Pinus caribaea* 6-7 years- Gallery Forest | 12,01 | 0,002 |
| *Pinus caribaea* 19-23 years- Gallery Forest | 12,55 | 0,002 |

**Appendix 4**. R Studio scripts used for the different data analyses

### BOOKSTORES###

library(readxl)

require(factoextra)

require (FactoMineR)

library(rcompanion)

library(multcompView)

library(tidyr)

library(dplyr)

library(vegan)

###PCA###

res.pca <- prcomp(JG1, scale = TRUE)

res.pca$rotation

res.pca$center

res.pca$scale

res.pca$x

fviz_eig(res.pca, main="",ylab="Porcentaje de varianza explicada",xlab="Dimensiones")

fviz_pca_ind(res.pca,

col.ind = "cos2", # Color by the quality of representation

gradient.cols = c("#00AFBB", "#E7B800", "#FC4E07"),

repel = TRUE # Avoid text overlapping

) + ggtitle("OBRERAS")

fviz_pca_var(res.pca, axes = c(1, 2),

col.var = "contrib", # Color by contributions to the PC

gradient.cols = c("#00AFBB", "#E7B800", "#FC4E07"),

repel = TRUE # Avoid text overlapping

)+ ggtitle("OBRERAS")

###KW###

kruskal.test(AN~COLONIA, cvc1)

PT = pairwise.wilcox.test(cvc1$AN,cvc1$COLONIA,

p.adjust.method="none")

PT = PT$p.value

PT1 = fullPTable(PT)

PT1

multcompLetters(PT1,

compare="<",

threshold=0.05,

Letters=letters,

reversed = F)

###CWM###

CWM1<-CWM_OBRERAS_2022

CWM1%>%

group_by(EDAD) %>%

summarize (La=weighted.mean(La, wts),

L1=weighted.mean(L1, wts),

L2=weighted.mean(L2, wts),

MPr=weighted.mean(MPr, wts),

Ra=weighted.mean(Ra, wts),

R1=weighted.mean(R1, wts),

R2=weighted.mean(R2, wts),

MP=weighted.mean(MP, wts),

Ind2=weighted.mean(Ind2, wts))

###PERMANOVA###

esp=vegdist(amb1,"manhattan")

adonis(esp~TRATAMIENTO,permutations = 999, amb1)

mod <- with(amb1, betadisper(esp,TRATAMIENTO))

permutest(mod)

source("parwise.adonis.txt")

pairwise.adonis(x = amb1, factors = ST$TRATAMIENTO,

sim.method ='manhattan', p.adjust.m = 'BH')

###NMDS###

windows()

plot(especies.nms2d, type="n", xlim=c(-0.5,0.5))

text(especies.nms2d$species, labels=row.names(especies.nms2d$species), cex=0.7)

ordiellipse(especies.nms2d,DS$TRATAMIENTO, scaling = "symmetric", col = "purple",

draw="polygon", show.groups = "2 AÑOS")

ordiellipse(especies.nms2d,DS$TRATAMIENTO, scaling = "symmetric", col = "blue",

draw="polygon", show.groups = "6 AÑOS")

ordiellipse(especies.nms2d,DS$TRATAMIENTO, scaling = "symmetric", col = "yellow",

draw="polygon", show.groups = "8 AÑOS")

ordiellipse(especies.nms2d,DS$TRATAMIENTO, scaling = "symmetric", col = "green",

draw="polygon", show.groups = "23 AÑOS")

ordiellipse(especies.nms2d,DS$TRATAMIENTO, scaling = "symmetric", col = "red",

draw="polygon", show.groups = "GALERÍA")

legend("topleft", legend=c("1-2 AÑOS", "6-7 AÑOS","7-8 AÑOS","19-23 AÑOS","GALERÍA"), pch=c(21,21),

pt.bg=c("purple", "blue", "yellow","green","red"), cex=0.7)
